# Acquired Lymphangioma Circumscriptum of the Scrotum: A Case Report

**DOI:** 10.7759/cureus.55895

**Published:** 2024-03-10

**Authors:** Marwah K Almalki, Alauldin K Alhowaish, Amer A Alharbi, Abdullah M Alsehli, Amin K Makhdoom

**Affiliations:** 1 Dermatology, King Fahad Hospital, Madinah, SAU; 2 Dermatology, King Khalid University Hospital, Riyadh, SAU; 3 Medicine and Surgery, Ohud Hospital, Madinah, SAU

**Keywords:** lesions, lymphatic vesicles, scrotum, lc, lymphangioma circumscriptum

## Abstract

Lymphangioma circumscriptum (LC) is an uncommon malformation affecting the skin and subcutaneous tissue. This report documents a case of LC that developed in the scrotum of a 35-year-old male. Upon examination, numerous clusters of clear vesicles were found on the scrotum's surface. The patient had no previous exposure to infections, trauma, surgery, or radiation treatment. A skin biopsy revealed enlarged lymphatic channels in the dermis, aligning with a diagnosis of LC. The patient was treated with cryotherapy using liquid nitrogen, specifically targeting the translucent yellowish vesicles. The cryotherapy was administered in a series of eight sessions, each involving double freeze-thaw cycles, spaced out at two-week intervals. Following treatment, the patient's lesions regressed, indicating a favorable therapeutic outcome. The patient was followed up for nearly one year, during which no new lesions developed, suggesting the treatment's effectiveness in preventing recurrence. The complete resolution of lesions and absence of recurrence during follow-up indicate a good prognosis and successful response to cryotherapy. Scrotal LC, particularly the acquired form in adults without any precipitating factors, is extremely rare. This case underlines the need to include acquired LC in the diagnostic considerations when adult patients present with vesicular lesions on the scrotum, to ensure accurate diagnosis and subsequent proper treatment.

## Introduction

Lymphangioma circumscriptum (LC), a rare dermatological condition, manifests as a proliferation of superficial lymphatic vesicles [1,2]. The incidence of LC is estimated at 1.2 to 2.8 cases per 100,000 people [1,2]. It is predominantly a pediatric ailment, often seen in the head and neck regions [1,2]. Scrotal involvement is particularly rare, with most cases being congenital and presenting early in life [2,3]. However, the acquired variant of LC, especially in the scrotal area, is a clinical anomaly that merits detailed exploration due to its rarity and diagnostic intricacies [4].

The pathogenesis of acquired LC involves lymphatic system disturbances commonly attributed to surgical procedures, radiation therapy, or infections leading to lymphatic damage or obstruction [5]. This pathophysiological mechanism is distinct from congenital LC, where the malformation is thought to stem from aberrant lymphatic tissue that fails to integrate into the normal lymphatic network during embryonic development [5,6].

This case report underscores an unusual presentation of acquired LC in the scrotal area of an adult male. Such presentations are scarce in medical literature, thereby making this case critical for enhancing our understanding of the condition. The report delves into the patient's clinical presentation, encompassing symptoms and lesion characteristics.

## Case presentation

**Figure 1:**
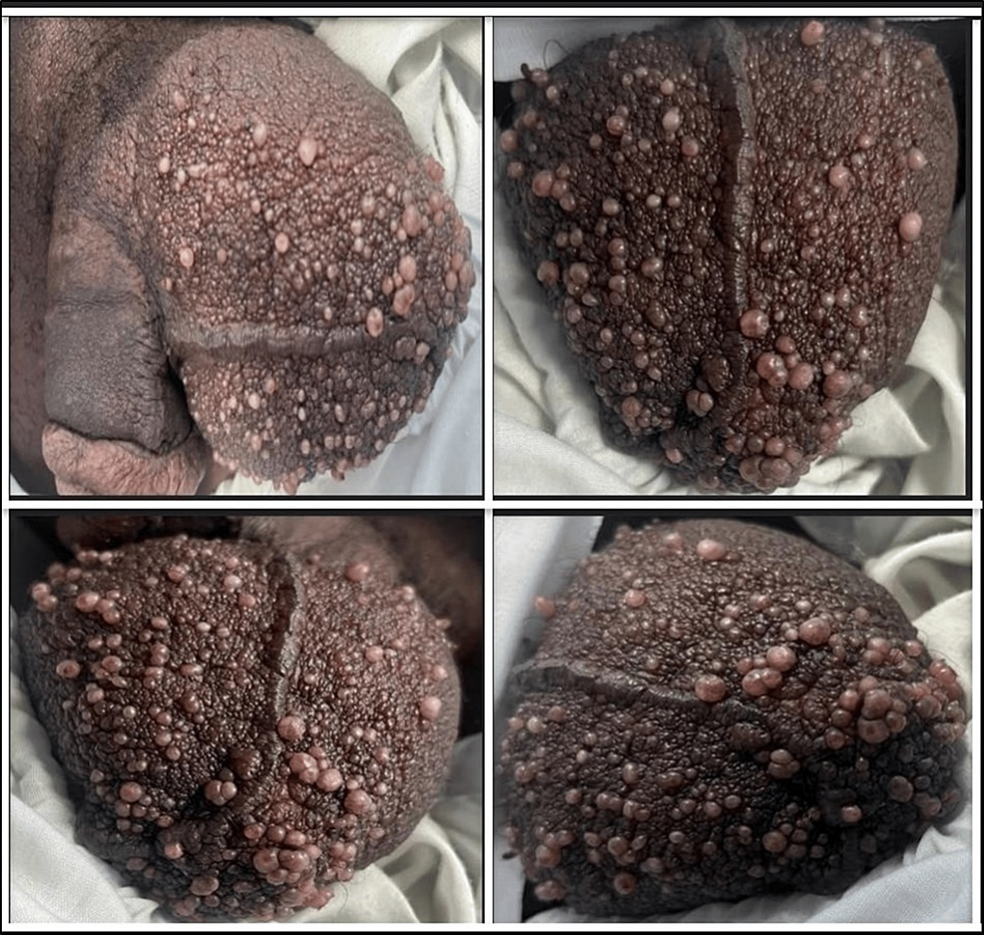
Clinical presentation of scrotal lymphangioma circumscriptum in this case

A 35-year-old Sudanese man presented to a dermatology clinic complaining of chronic, progressive, multiple groups of lesions on the scrotum that were painless, non-itchy, odorless, and were present for the past five years. On dermatological examination, the lesions show multiple erythematous clusters of varying-sized translucent vesicles that resembled frog spawn and involved almost the entire scrotal skin (Figure 1).

**Figure 2:**
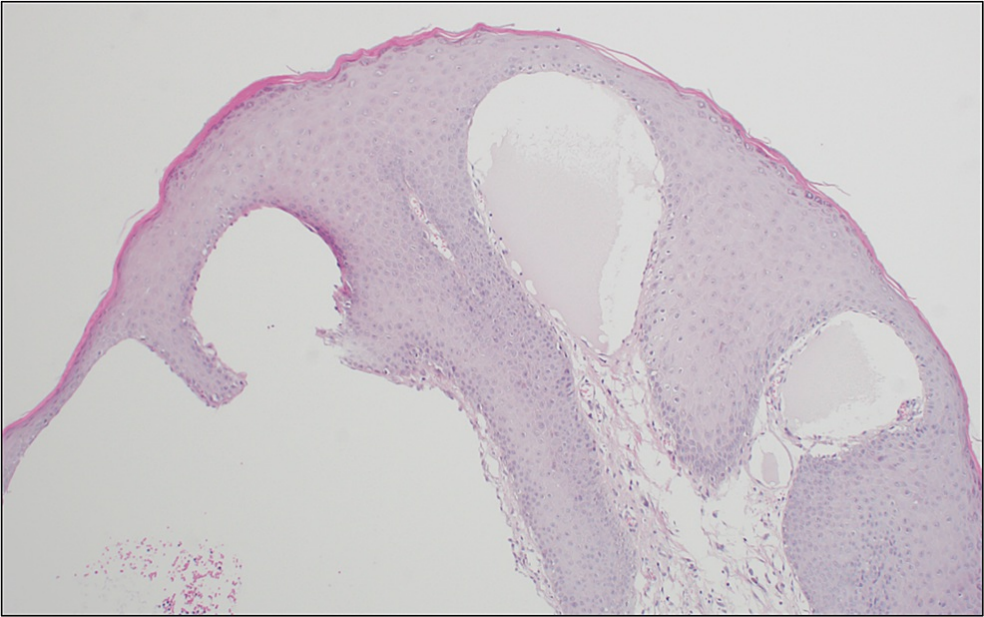
A skin biopsy confirming the diagnosis of lymphangioma circumscriptum

His medical history was notably free of any sexually transmitted diseases or a history of trauma or infection, and he had received neither surgery nor radiotherapy in the scrotal area. His laboratory profile did not show eosinophilia, and he was non-reactive for human immunodeficiency virus (HIV), hepatitis B surface antigen (HBs Ag), hepatitis C surface antigen (HCs Ag), or venereal disease research laboratory (VDRL). A skin biopsy was taken, and a microscopic examination showed acanthosis epidermis with underlying multiple thin-walled microcytic dilated lymphatic channels in the papillary dermis lined by flat endothelial cells and containing proteinaceous material. A diagnosis of LC was made (Figure 2). The patient was treated with cryotherapy using liquid nitrogen. The treatment was specifically targeted at the translucent yellowish vesicles that were characteristic of the patient's scrotal lymphangioma circumscriptum. The cryotherapy was administered in a series of eight sessions, each session involving double freeze-thaw cycles. These sessions were spaced out at regular two-week intervals to allow for adequate tissue recovery between treatments. The patient's response to cryotherapy was favorable. Following the completion of the treatment sessions, the patient's lesions were observed to have regressed, indicating a positive therapeutic outcome. To monitor for any potential recurrence or development of new lesions, the patient was followed up closely for a period of nearly one year after the conclusion of the cryotherapy sessions. During this follow-up period, the patient did not develop any new lesions on the scrotal skin. This suggests that cryotherapy treatment was effective in treating the existing lesions and preventing the development of new ones. The lack of recurrence over a considerable follow-up duration is a promising indicator of the treatment's success. Overall, the patient's prognosis appears to be good. The complete resolution of the lesions following cryotherapy and the absence of recurrence during the follow-up period suggest that the patient has responded well to the treatment.

## Discussion

Lymphangiomas are abnormal growths of the lymphatic vessels that can occur in a localized area or be more widespread throughout the body. Some theories suggest lymphangiomas are true neoplasms that arise when stromal or endothelial cells transform [4]. The pathogenesis of LC was first proposed by Whimster [7]. According to this theory, during embryonic development, numerous subdermal lymphatic cisterns form that do not connect with the lymphatic network [2,7]. These cisterns cannot drain lymph from surrounding tissues due to a lack of connection. The smooth muscle lining the cisterns contracts when compressed, causing the skin to protrude [2,7]. If the deep-collecting lymphatics are damaged by radiation, infections like filariasis, lymphogranuloma venereum, or tuberculosis and acquired LC can develop later in life [2,7].

Our patient had no prior radiation exposure or infections. LC of the scrotum is exceptionally rare, with very few cases described in the published medical literature. It most commonly occurs over the axillary folds, tongue, shoulders, neck, upper limbs, vulva, and buccal mucosa [7,8]. Clinically, LC can present as vesicles, nodules, or wart-like growths, thus making an accurate diagnosis challenging. The primary differential diagnoses are molluscum contagiosum, herpes zoster, anogenital warts, and leiomyomas. A definitive diagnosis requires a histopathological examination and biopsy [9]. In this case, a biopsy showed dilated lymphatic channels in the dermis lined by flat endothelium containing proteinaceous fluid, confirming the diagnosis of LC. Pathogenesis of acquired LC in adulthood without inciting factors, especially in anatomically uncommon sites like the scrotum, remains unclear. Proposed mechanisms include localized lymphatic damage from minimal trauma or possible somatic mutations in genes regulating lymphangiogenesis [10].

LC treatment options include surgical excision, laser ablation, skin fulguration by electrocautery or cryotherapy by liquid nitrogen, and other medical therapies like sirolimus or bleomycin [11,12]. Complete excision is preferred but has a high recurrence rate of up to 27% [11,12]. The rarity and diagnostic challenges of scrotal LC warrant increased clinical vigilance to facilitate accurate diagnosis and management. Further research is needed to elucidate the triggers for acquired LC presenting in adulthood in the absence of radiation, infection, trauma, or surgery.

## Conclusions

This case report documents an uncommon presentation of acquired LC in the scrotum of an adult male patient. Scrotal involvement of LC is rare, especially the acquired variant presenting in adulthood without inciting factors. This case highlights the importance of keeping acquired LC in the differential diagnosis when evaluating adult patients with a new onset of asymptomatic vesicular lesions on the scrotum. Increased awareness of this condition can help prevent misdiagnosis and allow for appropriate treatment. Further studies are needed to understand better the pathogenesis of acquired LC occurring in anatomically uncommon sites in adults without known triggers.
